# Liraglutide restores late cardioprotective effects of remote preconditioning in diabetic rats via activation of hydrogen sulfide and nuclear factor erythroid 2-related factor 2 signaling pathway

**DOI:** 10.1590/ACB360207

**Published:** 2021-02-26

**Authors:** Lingling Wang, Yinyan Tang, Huimin He, Weirong Wei

**Affiliations:** 1 MM. Jiangxi Provincial People’s Hospital – Department of Cardiology – Nanchang, (Jiangxi), China; 2 MM. The Forth People’s Hospital of Yongzhou – Department of Cardiovascular Medicine – Yangzhou (Hunan), China.; 3 MM. The Forth People’s Hospital of Yongzhou – Department of Cardiovascular Medicine – Yangzhou (Hunan), China.; 4 MM. Jiangxi Provincial People’s Hospital – Department of Cardiology – Nanchang, (Jiangxi), China.

**Keywords:** Liraglutide, Diabetes mellitus, Reperfusion injury, Hypoxia inducible factor, Langendorff

## Abstract

**Purpose:**

The present study explored the influence of liraglutide on remote preconditioning-mediated cardioprotection in diabetes mellitus along with the role of nuclear factor erythroid 2-related factor 2 (Nrf2), hypoxia inducible factor (HIF-1α) and hydrogen sulfide (H_2_S).

**Methods:**

Streptozotocin was given to rats to induce diabetes mellitus and rats were kept for eight weeks. Four cycles of ischemia and reperfusion were given to hind limb to induce remote preconditioning. After 24 h, hearts were isolated and subjected to 30 min of ischemia and 120 min of reperfusion on Langendorff system. Liraglutide was administered along with remote preconditioning. Cardiac injury was assessed by measuring the release of creatine kinase (CK-MB), cardiac troponin (cTnT) and development of left ventricular developed pressure. After ischemia-reperfusion, hearts were homogenized to measure the nuclear cytoplasmic ratio of Nrf2, H_2_S and HIF-1α levels.

**Results:**

In diabetic rats, there was more pronounced injury and the cardioprotective effects of remote preconditioning were not observed. Administration of liraglutide restored the cardioprotective effects of remote preconditioning in a dose-dependent manner. Moreover, liraglutide increased the Nrf2, H_2_S and HIF-1α levels in remote preconditioning-subjected diabetic rats.

**Conclusions:**

Liraglutide restores the lost cardioprotective effects of remote preconditioning in diabetes by increasing the expression of Nrf2, H_2_S and HIF-1α.

## Introduction

Diabetes mellitus is a chronic metabolic disorder, which is characterized by disturbance in glucose metabolism. Apart from short term effects of diabetes, long-standing uncontrolled hyperglycemia in diabetes produces a number of complications, including increase in the tendency to develop ischemia-reperfusion induced myocardial injury^1^,^2^. Remote preconditioning a therapeutic strategy in which short episodes of ischemia and reperfusion to an organ other than heart (nontarget or remote organ) confers protection to heart from sustained ischemia-reperfusion injury^3^. This therapeutic strategy has been used in preclinical as well in clinical setup to confer protection to heart against ischemia-reperfusion injury^4^,^5^. Amongst the different problems of long-standing diabetes, the usefulness of remote preconditioning to trigger cardioprotection is abolished significantly in diabetic condition^6^.

Liraglutide is glucagon-like peptide 1 agonist (GLP-1 agonist) and it has been used to manage diabetes mellitus type 2^7^. It is also been increasingly used to manage weight in obese people^8^with potential weight loss benefits, approved for the treatment of type 2 diabetes (T2D. Apart from it, liraglutide has been shown to exert neuroprotective effect and preserve cognitive functions^9^, decrease renal fibrosis^10^ and improve heart contractility^11^ and decrease heart remodeling^12^. There are important mediators revealed by different scientists that are important in inducing cardioprotective effects of remote preconditioning. Amongst these, the role of Nrf2 ratio^13^,^14^, HIF-1α^15^,^16^ and H_2_S^17^ has been very well documented in remote preconditioning-induced cardioprotection. Studies have shown that liraglutide promotes angiogenesis through increase in HIF-1α and vascular endothelial growth factor (VEGF) levels^18^. Moreover, it is also shown that liraglutide increases the expression of Nrf2, a transcriptional factor involved in increasing antioxidant enzymes, in producing beneficial effects^19^,^20^.

Based on these, the present study was designed to explore the influence of liraglutide on remote preconditioning-mediated cardioprotection in long standing diabetes mellitus. Moreover, the study also explored the possible involvement of Nrf2, HIF-1 and H_2_S in liraglutide-mediated effects in remote preconditioning-subjected diabetic rats.

## Methods

### Animals and drugs

The experimental protocol was approved by Institutional Ethical Committee of Jiangxi Provincial People’s Hospital, with ethic number: 202005088779C09. Wistar albino rats (200–250g) were used for this study and the animals were kept in the standardized laboratory facilities. The kits for estimating the levels of creatine kinase (CK-MB), cardiac troponin T (cTnT), HIF-1α and Nrf-2 were procured from MyBioSource, Inc., San Diego, CA, USA.

### Induction of diabetes mellitus

A single dose of streptozotocin (STZ) (60 mg/kg i.v.) was administered to rats to induce diabetes mellitus^21^. The animals were kept for eight weeks to allow the onset of diabetic complications including increase in the tendency to develop ischemia-reperfusion injury. The blood glucose levels were quantified before STZ injection and at the end of the 8^th^ week.

### Perfusion of isolated hearts on the Langendorff system and assessment of myocardial injury

The animals were sacrificed by cervical dislocation and hearts were isolated to perfuse on the Langendorff apparatus, using Krebs–Henseleit solution. The inflow of Krebs solution was stopped for 30 min to induce global ischemia. Thereafter, the flow was reinstated for reperfusion for 21 min^22^. The extent of ischemia-reperfusion injury was assessed by measuring the release of heart-specific CK-MB and cTnT from the heart to the coronary effluent using commercially available diagnostic kits. Moreover, the functional assessment of heart was evaluated by measuring left ventricular developed pressure (LVDP) in the heart using a pressure transducer.

### Remote ischemic preconditioning (RIP)

Under anesthesia (thiopental sodium 45 mg/kg i.p.), the left hind limb of rats was tied with neonatal blood pressure cuff. The cuff was filled with air up to pressure 160 mm of Hg to stop the flow of blood (hind limb ischemia) for 5 min. Thereafter, the cuff was deflated completely to restore the blood flow to hind limb (hind limb reperfusion) for 5 min. Such four alternate cycles of ischemia and reperfusion to the hind limb constituted remote preconditioning. After 24 h, the rats were sacrificed to isolate hearts, which were perfused on the Langendorff apparatus^3^.

### Assessment of nuclear cytoplasmic ratio of Nrf2, myocardial H_2_S and HIF-1α levels

After ischemia-reperfusion injury, the hearts were isolated for biochemical estimations. One half of the heart portion was homogenized in phosphate buffer saline (PBS, pH: 7.4). It was followed by centrifugation at 5000g for 15 min to obtain clear supernatant solution. The levels of H_2_S and HIF-1**α** were determined in clear supernatant solution. The levels of H_2_S were measured in the heart homogenates following ischemia-reperfusion injury using reverse phase high-performance liquid chromatography (HPLC) method and data were represented as μM/mg of protein. The levels of proteins in the heart homogenate were measured using Folin–Lowry method. The levels of HIF-1α were also determined in supernatant solution using ELISA kit. The other half portion of the heart was used to quantify nuclear cytoplasmic ratio of Nrf2. The nuclear and cytoplasmic fractions were separated using an extraction kit (BioVision, USA). The levels of Nrf2 were assessed in the supernatant using commercially available ELISA kits.

### Design

Nine experimental groups were used and each group comprised of eight rats. The groups included: normal control (group I), in which no intervention was done and heart was isolated for biochemical estimations; nondiabetic control (group II), in which hearts were isolated from nondiabetic rats and subjected to ischemia-reperfusion injury; diabetic control (group III), in which hearts of diabetic rats were subjected to ischemia-reperfusion injury; RIP in nondiabetic (group IV), in which RIP stimulus was given to nondiabetic rats; RIP in diabetic (group V), in which RIP stimulus was given to diabetic rats; liraglutide (0.2 mg/kg) and RIP in diabetic (group VI), in which liraglutide (0.2 mg/kg) was injected along with RIP stimulus in diabetic rats; liraglutide (0.4 mg/kg) and RIP in diabetic (group VII) in liraglutide (0.4 mg/kg) was injected along with RIP stimulus in diabetic rats; liraglutide (0.4 mg/kg) in nondiabetic control (group VIII), in which liraglutide was injected in nondiabetic animals, not subjected to RIP stimulus; liraglutide (0.4 mg/kg) and RIP in nondiabetic (group IX), in which liraglutide was injected along with RIP stimulus in nondiabetic rats.

### Statistical analysis

Mean± S.D. was used to represent the data. The data of CK-MB, cTnT, LVDP, blood glucose levels were statistically compared using two-way repeated measure ANOVA. The data of other parameters were compared using one-way ANOVA. Tukey’s *post hoc* test was employed for multiple comparisons. P < 0.05 was considered to be statistically significant.

## Results

### Effect of diabetes on ischemia-reperfusion induced heart injury

**Figure 1:**
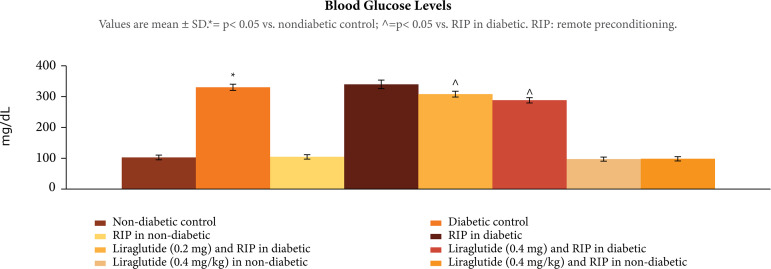
Effect of different interventions on blood glucose levels, assessed at the end of the 8th week.

**Figure 2:**
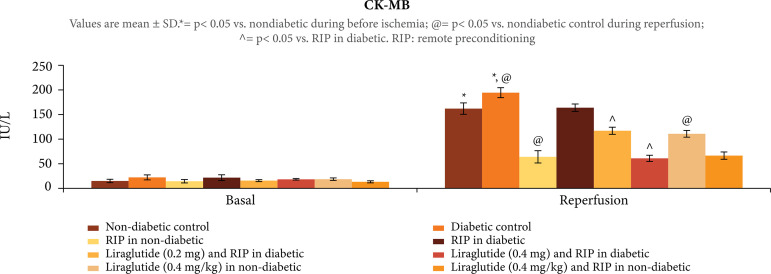
Effect of different interventions on the CK-MB levels, assessed in the coronary effluent before ischemia and during reperfusion.

**Figure 3:**
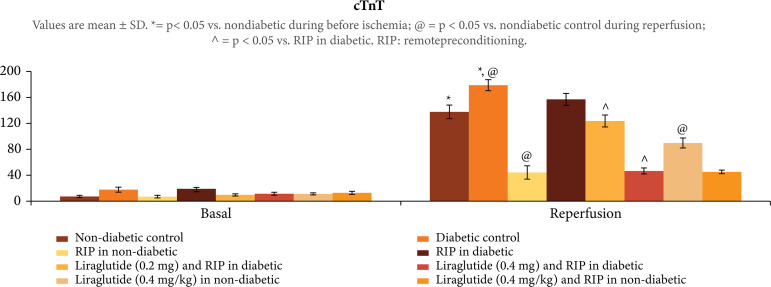
Effect of different interventions on the cTnT levels, assessed in the coronary effluent before ischemia and during reperfusion.

There was a significant rise in the fasting blood glucose levels in streptozotocin-injected rats as compared to nondiabetic rats, p < 0.001 (Fig. 1). The exposure of30 min of ischemia and 120 min of reperfusion to isolated rat hearts produced significant myocardial injury as assessed by increase in the release of CK-MB, p < 0.001 (Fig. 2) and cTnT, p < 0.001 (Fig. 3) in the coronary effluent. Moreover, there was also a significant decline in the functional parameters of heart assessed in terms of LVDP and there was a decrease in the value of LVDP during the reperfusion phase in comparison to pre-ischemic state (before ischemia), p < 0.001 (Table 1). In diabetic rats, there was a significant increase in heart injury in response to 30 min of ischemia and 120 min of reperfusion in comparison to normal, nondiabetic rats. In diabetic rats, there was higher release of CK-MB, p < 0.01 (Fig. 2), and cTnT, p < 0.01 (Fig. 3), in coronary effluent along with more depressed LVDP value, p < 0.01 (Table 1), in comparison to nondiabetic rats. It suggests that there was more significant myocardial injury in diabetic rats in comparison to nondiabetic rats.

**Table 1:** Effect of different interventions on the LVDP values, assessed before ischemia and during reperfusion.

S. No	Experimental	Before ischemia	During reperfusion
1.	Nondiabetic control	76.50 ± 3.54	33.50 ± 3.25*
2.	Diabetic control	64.10 ± 2.41	23.00 ± 1.77@
3.	RIP in nondiabetic	76.37 ± 2.38	55.75 ± 2.60@
4.	RIP in diabetic	63.75 ± 2.25	31.30 ± 2.19
5.	Liraglutide (0.2 mg) and RIP in diabetic	72.25 ± 2.31	45.15 ± 3.18^
6.	Liraglutide (0.4 mg) and RIP in diabetic	70.87 ± 2.29	58.00 ±3.25^
7.	Liraglutide (0.4 mg/kg) in nondiabetic	69.75 ± 2.37	39.80 ± 1.45@
8.	Liraglutide (0.4 mg/kg) and RIP in nondiabetic	70.50 ± 2.56	56.15 ± 3.44

Values are mean ± SD.

* = p < 0.05 vs. nondiabetic during before ischemia;

@ = p < 0.05 vs. nondiabetic control during reperfusion;

^ = p < 0.05 vs. RIP in diabetic. RIP: remote preconditioning; LVDP: left ventricular developed pressure.

### Effect of remote preconditioning onischemia-reperfusion induced injury in nondiabetic rats

Remote preconditioning stimulus produced significant delayed cardioprotection (assessed after 24 h of stimulus) in ischemia-reperfusion subjected nondiabetic rats. There was significant decline in CK-MB, p < 0.001 (Fig. 2), and cTnT, p < 0.001 (Fig. 3), levels along with improvement in LVDP values, p < 0.001 (Table 1), in remote preconditioning-subjected rats. However, the cardioprotective effects of remote preconditioning were not observed in streptozotocin-injected diabetic rats and there was no significant decrease in heart biomarkers or increase in heart contractility.

### Effects of liraglutide on remotepreconditioning-mediated actions in diabetic rats

Administration of liraglutide (0.2 and 0.4 mg/kg) in rats restored the cardioprotective effects of remote preconditioning as there was significant decline in heart injury biomarkers, p < 0.01 (Figs. 2 and 3), and improvement in heart contractility (Table 1) in a dose-dependent manner, p < 0.01. However, administration of liraglutide (0.4 mg/kg) in nondiabetic rats did not enhance the cardioprotection offered by remote preconditioning p > 0.05.

### Influence of different interventions on the biochemical parameters in ischemia-reperfusion subjected rats

**Figure 4:**
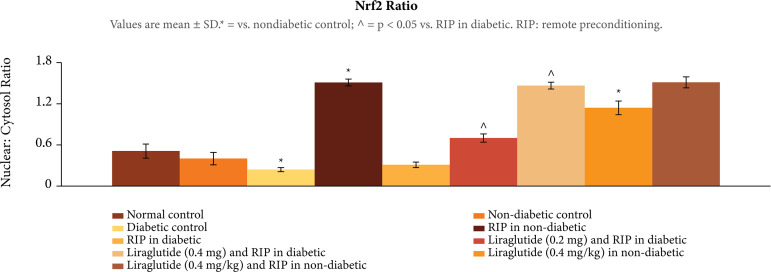
Effect of different interventions on the nuclear cytoplasmic Nrf2 ratio in the heart following ischemia-reperfusion injury.

**Figure 5:**
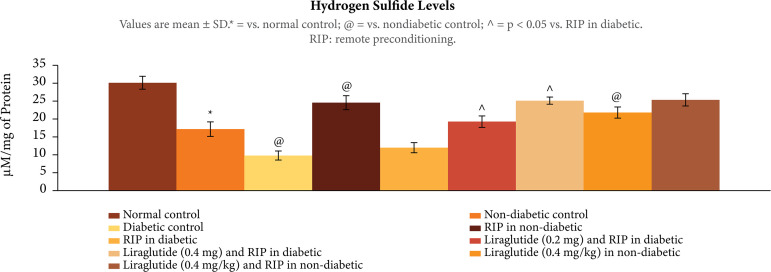
Effect of different interventions on the H2S levels in the heart following ischemia-reperfusion injury.

**Figure 6:**
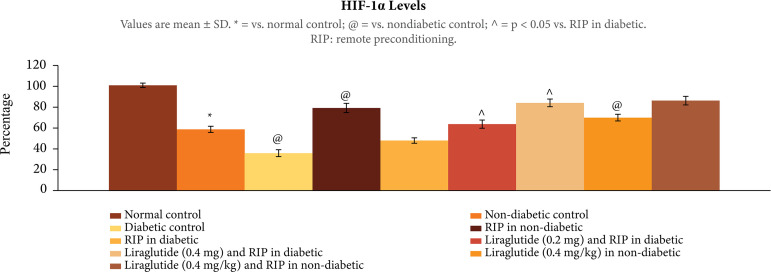
Effect of different interventions on the HIF-1α levels in the heart following ischemia-reperfusion injury.

In ischemia-reperfusion subjected rats, there was significant decrease in Nrf2 ratio, p < 0.01 (Fig. 4), H2S, p < 0.01 (Fig. 5), and HIF-1α levels, p < 0.01 (Fig. 6), in the heart homogenates. The decrease in these biochemical parameters was more prominent in diabetic rats. Remote preconditioning restored the Nrf2 ratio, H2Sand HIF-1α levels selectively in nondiabetic rats (p < 0.01), without any significant effect in diabetic rats p > 0.05. Administration of liraglutide (0.2 and 0.4 mg/kg) restored the effects of remote preconditioning and there was a significant increase in Nrf2 ratio, H2Sand HIF-1α levels, p < 0.01. Liraglutide did not enhance the effects of remote preconditioning on biochemical parameters, p > 0.05.

## Discussion

In the present investigation, ischemia-reperfusion produced a significant injury as assessed by an increase in CK-MB and cTnT release along with a decrease in LVDP. The extent of myocardial injury was much higher in diabetic rats in comparison to nondiabetic rats. The present study results showing an increase in myocardial injury in diabetic rats in response to ischemia-reperfusion injury is in consonance with the previous studies^23^,^24^. In the present study, remote preconditioning attenuated ischemia-reperfusion injury myocardial injury, which was assessed after 24 h of remote preconditioning stimulus, suggesting that remote preconditioning produced delayed cardioprotective effects in nondiabetic rats. However, the cardioprotective effects of remote preconditioning were not observed in nondiabetic rats. In other words, remote preconditioning failed to attenuate ischemia-reperfusion induced myocardial injury in diabetic rats. The present study results showing the attenuated protective effects of remote preconditioning in diabetic rats are in consonance with previously published studies^6^,^25^. It has been suggested that long standing diabetes mellitus interferes with the endogenous protective mechanism and loss of protective mechanism may contribute to an increase in ischemia-reperfusion injury.

In this study, administration of liraglutide (0.2 mg and 0.4 mg/kg) led to restoration of cardioprotective effects of remote preconditioning and in liraglutide-treated rats, remote preconditioning attenuated ischemia-reperfusion injury in a significant manner in diabetic rats. Liraglutide is a glucagon-like peptide 1 agonist and is used in the management of diabetes mellitus^8^,^26^. Apart from its antidiabetic action, it has been shown to produce beneficial effect on heart, brain and other organs^9^,^27^,^28^,^29^. It is reported to repair the infarcted heart^30^, produce beneficial effects on heart failure^31^. Nevertheless, it is the first study describing the usefulness of liraglutide in restoring the lost late cardioprotective effects of remote preconditioning in diabetic rats.

In the present study, there were significant biochemical alterations in the heart of ischemia-reperfusion subjected rats. There was a significant decrease in the nuclear cytosolic ratio of Nrf2 along with a decrease in the levels of H_2_S and HIF-1α in the hearts of ischemia-reperfusion subjected rats. These biochemical alterations were significantly more pronounced in diabetic rats, suggesting that long-term standing hyperglycemia may have adversely affected these biochemical parameters, which may be responsible for increase in myocardial injury in diabetic rats. Interestingly, remote preconditioning-induced cardioprotection was associated with the increase in the levels of Nrf2, H_2_S and HIF-1α in the hearts of ischemia-reperfusion subjected rats. There have been studies showing the key role of Nrf2^14^, H_2_S^17^ and HIF-1α^32^ in remote preconditioning-induced cardioprotection in ischemia-reperfusion subjected rats. However, remote preconditioning failed to increase the levels of these biochemical parameters in diabetic rats, suggesting that failure to increase the levels of Nrf2, H_2_Sand HIF-1α in diabetic rats may be a possible mechanism contributing in attenuating the beneficial effects of remote preconditioning.

In the present study, liraglutide-mediated restoration of cardioprotective effects of remote preconditioning in diabetic rats was associated with a significant increase in the levels of Nrf2, H_2_S and HIF-1α in the heart homogenates. Hypoxia inducible factor is a hypoxia-inducible transcriptional factor, which is involved in producing beneficial effects on the heart by virtue of multiple mechanisms, including increase in angiogenesis^33^. There has also been a study showing that liraglutide promotes angiogenesis by increasing the levels of HIF-1α^18^. Moreover, it is also shown that liraglutide increases the Nrf2 ratio to exert antioxidant effects^34^. However, it is the first study showing the increase in the H_2_S levels following liraglutide treatment in diabetic rats. Based on the results of the present study, it may be proposed that liraglutide may restore the cardioprotective effects of remote preconditioning in diabetic rats by increasing the expression of Nrf2, H_2_S and HIF-1α.

## Conclusion

Liraglutide has the potential to restore the lost cardioprotective effects of remote preconditioning in diabetic rats by increasing the expression of Nrf2, H_2_S and HIF-1α.

## References

[1] Chatham JC, Young ME, Zhang J. (2020). Role of O-linked N-acetylglucosamine (O-GlcNAc) modification of
proteins in diabetic cardiovascular complications. Curr Opin Pharmacol.

[2] Napoli R, Formoso G, Piro S, Targher G, Consoli A, Purrello F. (2020). Management of type 2 diabetes for prevention of cardiovascular
disease. An expert opinion of the Italian Diabetes Society. Nutr Metab Cardiovasc Dis.

[3] Singh H, Kumar M, Singh N, Jaggi AS (2019). Late Phases of Cardioprotection During Remote Ischemic
Preconditioning and Adenosine Preconditioning Involve Activation of
Neurogenic Pathway. J Cardiovasc Pharmacol.

[4] Kosiuk J, Langenhan K, Hindricks G, Bollmann A, Dagres N. (2021). Remote ischemic preconditioning in a setting of electrical
cardioversion of early onset persistent atrial fibrillation (RIP CAF trial):
Rationale and study design. J Cardiol.

[5] Sawashita Y, Hirata N, Yoshikawa Y, Terada H, Tokinaga Y, Yamakage M. (2020). Remote ischemic preconditioning reduces myocardial
ischemia-reperfusion injury through unacylated ghrelin-induced activation of
the JAK/STAT pathway. Basic Res Cardiol.

[6] Tyagi S, Singh N, Virdi JK, Jaggi AS. (2019). Diabetes abolish cardioprotective effects of remote ischemic
conditioning: evidences and possible mechanisms. J Physiol Biochem.

[7] Rodrigues T, Borges P, Mar L, Marques D, Albano M, Eickhoff H, Carrêlo C, Almeida B, Pires S, Abrantes M, Martins B, Uriarte C, Botelho F, Gomes P, Silva S, Seiça R, Matafome P. (2020). GLP-1 improves adipose tissue glyoxalase activity and
capillarization improving insulin sensitivity in type 2
diabetes. Pharmacol Res.

[8] Mirabelli M, Chiefari E, Caroleo P, Arcidiacono B, Corigliano DM, Giuliano S, Brunetti FS, Tanyolaç S, Foti DP, Puccio L, Brunetti A. (2019). Long-Term Effectiveness of Liraglutide for Weight Management and
Glycemic Control in Type 2 Diabetes. Int J Environ Res Public Health.

[9] Yan W, Pang M, Yu Y, Gou X, Si P, Zhawatibai A, Zhang Y, Zhang M, Guo T, Yi X, Chen L. (2019). The neuroprotection of liraglutide on diabetic cognitive deficits
is associated with improved hippocampal synapses and inhibited neuronal
apoptosis. Life Sci..

[10] Ougaard ME, Sembach FE, Jensen HE, Pyke C, Knudsen LB, Kvist PH. (2020). Liraglutide improves the kidney function in a murine model of
chronic kidney disease. Nephron.

[11] Sassoon DJ, Tune JD, Mather KJ, Noblet JN, Eagleson MA, Conteh AM, Sturek JT, Goodwill AG. (2017). Glucagon-Like Peptide 1 Receptor Activation Augments Cardiac
Output and Improves Cardiac Efficiency in Obese Swine After Myocardial
Infarction. Diabetes.

[12] Zheng R-H, Bai X-J, Zhang W-W, Wang J, Bai F, Yan CP, James EA, Bose HS, Wang N-P, Zhao Z-Q. (2019). Liraglutide attenuates cardiac remodeling and improves heart
function after abdominal aortic constriction through blocking angiotensin II
type 1 receptor in rats. Drug Des Dev Ther.

[13] Herajärvi J, Anttila T, Dimova EY, Laukka T, Myllymäki M, Haapanen H, Olenchock BA, Tuominen H, Puistola U, Karihtala P, Kiviluoma K, Koivunen P, Anttila V, Juvonen T. (2017). Exploring effects of remote ischemic preconditioning in a pig
model of hypothermic circulatory arrest. Scand Cardiovasc J..

[14] Zhou C, Li H, Yao Y, Li L. (2014). Delayed Remote Ischemic Preconditioning Produces an Additive
Cardioprotection to Sevoflurane Postconditioning Through an Enhanced Heme
Oxygenase 1 Level Partly Via Nuclear Factor Erythroid 2-Related Factor 2
Nuclear Translocation. J Cardiovasc Pharmacol Ther.

[15] Heyman SN, Leibowitz D, Levi IM-Y, Liberman A, Eisenkraft A, Alcalai R, Khamaisi M, Rosenberger C. (2016). Adaptive response to hypoxia and remote ischaemia
pre‐conditioning: a new hypoxia‐inducible factors era in clinical
medicine. Acta Physiol (Oxf).

[16] Kalakech H, Tamareille S, Pons S, Godin-Ribuot D, Carmeliet P, Furber A, Martin V, Berdeaux A, Ghaleh B, Prunier F (2013). Role of hypoxia inducible factor-1α in remote limb ischemic
preconditioning. J Mol Cell Cardiol.

[17] Andreadou I, Iliodromitis EK, Rassaf T, Schulz R, Papapetropoulos A, Ferdinandy P. (2015). The role of gasotransmitters NO, H2S and CO in myocardial
ischaemia/reperfusion injury and cardioprotection by preconditioning,
postconditioning and remote conditioning. Br J Pharmacol.

[18] Langlois A, Mura C, Bietiger W, Seyfritz E, Dollinger C, Peronet C, Maillard E, Pinget M, Jeandidier N, Sigrist S. (2016). *In Vitro* and *In Vivo* Investigation of the
Angiogenic Effects of Liraglutide during Islet
Transplantation. PLoS One.

[19] Zhu C-G, Luo Y, Wang H, Li J-Y, Yang J, Liu Y-X, Qu H-Q, Wang B-L, Zhu M. (2020). Liraglutide Ameliorates Lipotoxicity-Induced Oxidative Stress by
Activating the NRF2 Pathway in HepG2 Cells. Horm Metab Res.

[20] Han X, Ding C, Zhang G, Pan R, Liu Y, Huang N, Hou N, Han F, Xu W, Sun X. (2020). Liraglutide ameliorates obesity-related nonalcoholic fatty liver
disease by regulating Sestrin2-mediated Nrf2/HO-1 pathway. Biochem Biophys Res Commun.

[21] Adams DM, Yakubu MT. (2020). Aqueous extract of *Digitaria exilis* grains
ameliorate diabetes in streptozotocin-induced diabetic male Wistar
rats. J Ethnopharmacol.

[22] Watanabe M, Okada T. (2018). Langendorff Perfusion Method as an Ex Vivo Model to Evaluate
Heart Function in Rats. Methods Mol Biol.

[23] Zhao D, Yang J, Yang L. (2017). Insights for Oxidative Stress and mTOR Signaling in Myocardial
Ischemia/Reperfusion Injury under Diabetes. Oxid Med Cell Longev.

[24] Xu G, Zhao X, Fu J, Wang X. (2019). Resveratrol increase myocardial Nrf2 expression in type 2
diabetic rats and alleviate myocardial ischemia/reperfusion injury
(MIRI). Ann Palliat Med.

[25] Wider J, Undyala VVR, Whittaker P, Woods J, Chen X, Przyklenk K. (2018). Remote ischemic preconditioning fails to reduce infarct size in
the Zucker fatty rat model of type-2 diabetes: role of defective humoral
communication. Basic Res Cardiol.

[26] le Roux, Astrup A, Fujioka K, Greenway F, Lau DCW, Van Gaal, Ortiz RV, Wilding JPH, Skjøth TV, Manning LS, Pi-Sunyer X (2017). 3 years of liraglutide versus placebo for type 2 diabetes risk
reduction and weight management in individuals with prediabetes: a
randomised, double-blind trial. Lancet.

[27] Li Y, Bader M, Tamargo I, Rubovitch V, Tweedie D, Pick CG, Greig NH (2015). Liraglutide is neurotrophic and neuroprotective in neuronal
cultures and mitigates mild traumatic brain injury in mice. J Neurochem.

[28] Li Y-K, Ma D-X, Wang Z-M, Hu X-F, Li S-L, Tian H-Z, Wang M-J, Shu Y-W, Yang J. (2018). The glucagon-like peptide-1 (GLP-1) analog liraglutide attenuates
renal fibrosis. Pharmacol Res.

[29] Ekström K, Dalsgaard M, Iversen K, Pedersen-Bjergaard U, Vejlstrup N, Diemar SS, Idorn M, Thorsteinsson B, Engstrøm T (2017). Effects of liraglutide and ischemic postconditioning on
myocardial salvage after I/R injury in pigs*. Scand Cardiovasc J.

[30] Qiao H, Ren H, Du H, Zhang M, Xiong X, Lv R. (2018). Liraglutide repairs the infarcted heart: The role of the
SIRT1/Parkin/mitophagy pathway. Mol Med Rep.

[31] Zhang Y, Chen Q, Huang G, Wang L. (2019). The influence of liraglutide for heart failure: a meta-analysis
of randomized controlled trials. Heart Surg Forum.

[32] Cai Z, Luo W, Zhan H, Semenza GL. (2013). Hypoxia-inducible factor 1 is required for remote ischemic
preconditioning of the heart. Proc Natl Acad Sci USA.

[33] Semenza GL (2014). Hypoxia-Inducible Factor 1 and Cardiovascular
Disease. Annu Rev Physiol.

[34] Deng C, Cao J, Han J, Li J, Li Z, Shi N, He J. (2018). Liraglutide Activates the Nrf2/HO-1 Antioxidant Pathway and
Protects Brain Nerve Cells against Cerebral Ischemia in Diabetic
Rats. Comput Intel lNeurosci.

